# Fabrication of High Aspect Ratio Micro-Structures with Superhydrophobic and Oleophobic Properties by Using Large-Area Roll-to-Plate Nanoimprint Lithography

**DOI:** 10.3390/nano11020339

**Published:** 2021-01-29

**Authors:** Nithi Atthi, Marc Dielen, Witsaroot Sripumkhai, Pattaraluck Pattamang, Rattanawan Meananeatra, Pawasuth Saengdee, Oraphan Thongsook, Norabadee Ranron, Krynnaras Pankong, Warinrampai Uahchinkul, Jakrapong Supadech, Nipapan Klunngien, Wutthinan Jeamsaksiri, Pim Veldhuizen, Jan Matthijs ter Meulen

**Affiliations:** 1 Thai Microelectronics Center (TMEC), National Electronics and Computer Technology Center (NECTEC), Chachoengsao 24000, Thailand; witsaroot.sripumkhai@nectec.or.th (W.S.); pattaraluck.pattamang@nectec.or.th (P.P.); rattanawan.meananeatra@nectec.or.th (R.M.); pawasuth.saengdee@nectec.or.th (P.S.); oraphan.thongsook@nectec.or.th (O.T.); norabadee.ran@nectec.or.th (N.R.); krynnaras.pan@ncr.nstda.or.th (K.P.); warinrampai.uah@ncr.nstda.or.th (W.U.); jakrapong.supadech@nectec.or.th (J.S.); nipapan.klunngien@nectec.or.th (N.K.); wutthinan.jeamsaksiri@nectec.or.th (W.J.); 2 Morphotonics B.V., De Run 4281, 5503 LM Veldhoven, The Netherlands; marc.dielen@morphotonics.com (M.D.); pim.veldhuizen@morphotonics.com (P.V.); jan.matthijs.ter.meulen@morphotonics.com (J.M.t.M.)

**Keywords:** high aspect ratio micro-structure, roll-to-plate nanoimprint lithography, superhydrophobic, oleophobic, biomimetic surface, large-area patterning

## Abstract

Bio-inspired surfaces with superamphiphobic properties are well known as effective candidates for antifouling technology. However, the limitation of large-area mastering, patterning and pattern collapsing upon physical contact are the bottleneck for practical utilization in marine and medical applications. In this study, a roll-to-plate nanoimprint lithography (R2P NIL) process using Morphotonics’ automated Portis NIL600 tool was used to replicate high aspect ratio (5.0) micro-structures via reusable intermediate flexible stamps that were fabricated from silicon master molds. Two types of Morphotonics’ in-house UV-curable resins were used to replicate a micro-pillar (PIL) and circular rings with eight stripe supporters (C-RESS) micro-structure onto polycarbonate (PC) and polyethylene terephthalate (PET) foil substrates. The pattern quality and surface wettability was compared to a conventional polydimethylsiloxane (PDMS) soft lithography process. It was found that the heights of the R2P NIL replicated PIL and C-RESS patterns deviated less than 6% and 5% from the pattern design, respectively. Moreover, the surface wettability of the imprinted PIL and C-RESS patterns was found to be superhydro- and oleophobic and hydro- and oleophobic, respectively, with good robustness for the C-RESS micro-structure. Therefore, the R2P NIL process is expected to be a promising method to fabricate robust C-RESS micro-structures for large-scale anti-biofouling application.

## 1. Introduction

Biofouling from colonization of various organisms, pathogens and inorganic macromolecules (mostly proteins) is the unwanted accumulation of biological and inorganic matters on wetted surfaces [1]. The contamination of surfaces, such as marine infrastructure, medical devices and other engineering components, has been a global issue with significant impact on the environment, health risks and economics [1,2]. Biofouling by numerous pathogens such as viruses, bacteria, fungi and other infectious agents causes a spread of infectious diseases in public space which potentially leads to thousands of annual deaths worldwide [1]. Being submerged in seawater, surfaces of a ship hull are exposed to thousands of species of fouling organisms such as barnacles, mussels and seaweed. Issues include marine corrosion and increased ship hull drag, which result in reduced speed, increased fuel consumption and emissions of greenhouse gases (CO_2_, NO_x_, and SO_2_) [3]. On top of that, it costs the global economy USD 150 billion yearly due to the environmental and significant economic impacts [2,3].

For antibacterial and antifouling solutions in medical environments, such as a surgery room and ward area, expensive materials such as Teflon and paint with nanoparticles (NPs) are commonly used. However, the processes to fabricate silver (Ag), titanium dioxide (TiO_2_) and zinc oxide (ZnO) NPs as well as the manufacturing and the spray coating of the paint with non-bonded NPs can be toxic if not taking careful precautionary measures [4,5,6]. In 2008, the use of biocide-based paint that contains toxic substances such as Tributhyltin (TBT) and lead (Pb) was banned because of its severe effect on living organisms in the ocean [7]. Subsequently, non-biocide-release approaches, such as water jet cleaning and ultrasonic wave methods, were proposed. However, these technologies are very harmful for the operator working in underwater environment. Moreover, the ultrasonic waves can interfere the communication of marine mammals [8,9]. Therefore, alternative methods that are inexpensive, robust, easy to apply, scalable, and environmentally friendly are required. Super dewetting or antifouling surfaces have shown excellent resistance to stains, bacteria, proteins, and various marine organisms due to the absence of effective adhesion points on these surfaces.

The super dewetting surfaces exhibit good anti-biofouling and self-cleaning properties, which have the potential to address the concerns of the above-mentioned traditional biocide-based antifouling methods. There are three types of antifouling surfaces: superhydrophobic surfaces, underwater superoleophobic surfaces, and slippery liquid-infused porous surfaces (SLIPS). Among these, superhydrophobic surfaces, with a contact angle of water (surface tension, *γ_lv_* = 72.1 mN/m) greater than 150° and a sliding angle smaller than 10°, are one of the promising technologies for antifouling purpose [8,9,10,11,12]. Such superhydrophobic surfaces are often inspired by nature. For instance, it is reported that the surfaces of various natural animals and plants, such as lotus leaves, red rose petals, butterfly wings, mosquito eyes, Salvinia leaves, water strider legs, gecko feet, and shark skin, show exceptional wetting behavior with superhydrophobicity [13,14,15,16,17,18].

**Figure 1:**
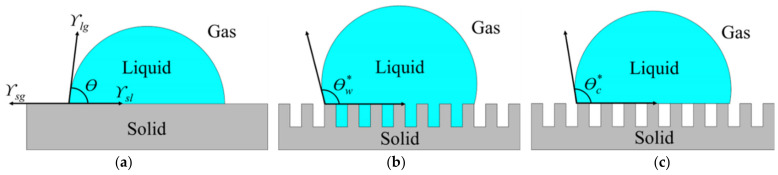
A liquid droplet resting on a solid surface and surrounded by a gas forms a characteristic contact angle *θ*. (**a**) Flat surface; (**b**) Rough surface at which the liquid is in intimate contact with the solid asperities, so that the droplet is in the Wenzel state; (**c**) Rough surface at which the liquid rests on top of the asperities, so that the droplet is in the Cassie-Baxter state [20,21]. Note that θ_w_* is the apparent contact angle of the textured surface based on the Wenzel model, θ_c_* is the apparent contact angle of the textured surface based on the Cassie-Baxter model, and *γ_sv_*, *γ_sl_*, *γ_lv_* is the surface tension between solid-gas, solid-liquid, and liquid-gas interfaces, respectively.

Basically, surface wettability is categorized by the contact angle of the liquid droplet [19,20]. Firstly, if the water contact angle (WCA) is smaller than 90°, a surface is considered hydrophilic. Secondly, if the WCA is greater than 90°, it is considered hydrophobic. Lastly, if the WCA is greater than 150°, it is considered superhydrophobic [21]. Typically, the contact angle of liquid droplets on a flat surface can be explained by Young’s model as shown in Figure 1a. However, a surface is never completely smooth and generally exhibits a surface roughness. The effect of surface roughness on the apparent contact angle of liquid droplets can be explained by the Wenzel and Cassie-Baxter models as shown in Figure 1b,c, respectively [22].

The Wenzel model is used to describe the wetting behavior of a rough surface by calculating the apparent contact angle of the textured surface (θ_w_*) based on its surface roughness (*r*), surface tension between solid–gas (*γ_sv_*), solid–liquid (*γ_sl_*), and liquid–gas (*γ_lv_*) interfaces, and an intrinsic contact angle of the liquid droplet on a flat surface of the same material (*θ*), as shown in Equation (1) [22,23,24]. In the Wenzel state, the liquid wets the surface and completely fills all voids on the rough surface. The contact angle in the Wenzel state is defined by:

(1) $$\cos\theta_{W}*=\frac{r\left(\gamma_{sv}-\gamma_{sl}\right)}{\gamma_{lv}}=r\cos\theta$$

in which the surface roughness factor r is defined by the ratio of the actual surface area of the rough surface (*A_h_*) to the projected area (*A_f_*), as shown in Equation (2):

(2) $$r=1+\frac{A_{h}}{A_{f}}$$

The Cassie-Baxter model describes that the ultimate liquid-repellent nature of the rough surface is caused by microscopic air pockets filled in the space between the rough micro- or nano-structures and the liquid droplet. The air pockets then create a combination of air–liquid–solid interfaces [13,21,25]. If *ϕ_s_* is the fraction of the solid in contact with the liquid, the Cassie-Baxter equation can be expressed as Equation (3).

(3) $$\cos\theta_{C}{}^{*}=-1+\phi_{s}(1+\cos\theta )$$

**Figure 2:**
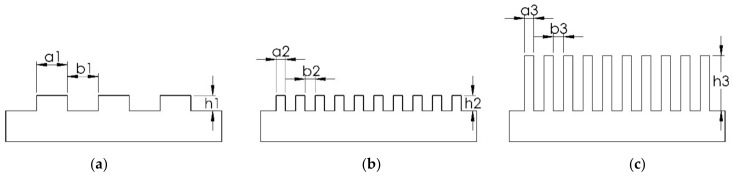
Schematic diagram of an example micro-/nano-structure having a different surface roughness (*r*) due to a different packing factor (P = a/b) and aspect ratio (A.R. = h/a), where a is the pattern width, b is the pattern spacing, and h is the pattern height. (**a**) Low P and low A.R., (**b**) high P and low A.R., and (**c**) high P and high A.R. pattern.

To maximize the WCA in Wenzel’s model, Young’s contact angle (*θ*), as determined on a flat surface, has to be minimized and the surface roughness (*r*) has to be maximized [26]. There are two methods to construct a superhydrophobic surface. The first one is to reduce the surface energy of the material, and the other is to increase the surface roughness. In the past decades, many research groups designed different hierarchical micro-/nano-structures on low surface energy material to produce a biomimetic superhydrophobic surface. The value of *r* of the patterned surface has to be maximized by reducing the pattern width (a) and pattern spacing (b) down to the nanometer scale, and by increasing the pattern height (h) [27,28,29]. This is shown schematically in Figure 2a–c, respectively, in which the surface roughness *r* of an example micro-/nano-structure is increased by increasing the packing factor (P = a/b) and the aspect ratio (A.R. = h/a).

Note that here the pattern width is equal to the pattern spacing (a_1_ = b_1_, a_2_ = b_2_, a_3_ = b_3_) but where a_1_ < (a_2_ = a_3_) and (h_1_ = h_2_) < h_3_. The highest packing factor can be achieved with the smallest surface structures (i.e., nano-structures). These are typically made in a conventional lithography process. In making such silicon (Si) master molds, the resolution of the lithographic exposure tool and performance of the deep reactive ion etching (DRIE) tool limits a maximum value of the packing factor and a maximum value of the aspect ratio of the hierarchical micro-/nano-structures.

As structured substrate material, Polydimethylsiloxane (PDMS) is commonly known for its low surface energy (*γ_sv_* = 12–16 mJ/m^2^), low Young’s modulus (∼2.0 MPa), good thermal and oxidative stability, non-toxicity, good biocompatibility, and low cost. PDMS also provides a conformal contact and it is released easily from a Si master mold [30,31]. Moreover, PDMS has a great degree of flexibility during the patterning process due to its relatively high toughness and high elongation at break (>150%) [32,33]. Based on all of these benefits combined, PDMS becomes one of the best material choices that is widely used to fabricate surfaces with superhydrophobic properties using a soft lithography process [32,34,35]. A flat PDMS surface only shows hydrophobic properties with a WCA of 107–110° [36]. Therefore, the fabrication of a rough surface with engineered hierarchical micro-/nano-structures having controlled geometries by the soft lithography process is needed to make PDMS become superhydrophobic [33,34]. Regarding our previous work, PDMS surfaces patterned with a conventional square-like pillar pattern arranged in a square array can demonstrate superhydrophobicity (WCA > 150°) when *r* and A.R. are greater than 2.75 and 3.0, respectively [27]. To obtain a superoleophobic and superamphiphobic surface, the A.R. of the micro-/nano-structures must even be greater than 5.0.

Typically, there are two ways to create a nature-inspired superhydrophobic surface with hierarchical micro-/nano-structured rough surface on a substrate with low surface energy: replication of a biological surface by a casting (i.e., soft lithography) process or an engineered rigid master template in a “top-down approach” photolithographic method. The soft lithography process is widely studied because it is easy and accurate to replicate the micro-/nano-patterns with various A.R. on the relatively soft PDMS substrate. However, the flexibility of PDMS (σ: 5.0 MPa, ε: 116% @RT) limits its feasibility in the processability of high A.R. structures and increasing pattern density, hydrophobicity, and mechanical strength. The challenges of using PDMS also include deformation, merging, and collapsing of structures, which result in decreased hydrophobicity [35].

Therefore, there are three main approaches to improve the robustness and strength of these superhydrophobic surfaces. Firstly, a well-designed robust micro-/nano-structure is required to prevent the PDMS pattern from collapsing and maintain its superhydrophobicity [36]. Secondly, utilization of PDMS-based composite materials to improve its mechanical properties [37,38]. Note that the surface energy of the PDMS-based composite materials is often higher, which adversely affects the superhydrophobic properties. Thirdly, development of new materials, besides PDMS, with low surface energy and high mechanical strength.

To realize the full potential of antifouling surfaces, engineered hierarchical micro-/nano-structures with superhydrophobic and superoleophobic properties need to be produced over a large area in a cost-efficient manner for practical applications [39]. This continuous and efficient fabrication of superhydrophobic surfaces on large areas is challenging and few processes have been adopted by industrial concerns. This is due to the fact that most of the reported methods require multiple complex processing steps, and have low throughput, substrate limitations, or high production cost [40,41,42,43,44]. Among many conventional micro- and nano-patterning techniques that have been developed in the past few decades, the roll-to-plate ultraviolet (UV) nanoimprint lithography (R2P NIL) and the roll-to-roll UV nanoimprint lithography (R2R NIL) technologies are some of the promising solutions due to the lower cost, higher throughput, larger patterning area, and higher resolution beyond the limitations set by light diffraction or beam scattering that are encountered in other traditional micro-/nano-fabrication techniques [44].

One of the technical challenges of the UV NIL process is the scaling of the master. As the patterns have to be transferred from a Si master mold (or other master material) in order to fabricate a flexible stamp that can be reused, the fabrication of large-area Si molds tends to be difficult as the feature sizes go down to lower ranges of the nanometer scale. Therefore, often a scaling up of a small area on a (expensive) master mold containing the micro- or nano-structures needs to be performed. Additionally, the material selection for the UV NIL process is also crucial in overcoming critical issues such as the well-known stamp sticking problem, polymerization shrinkage, and thermal and hygroscopic expansion as well as prolonged lifetime of the flexible stamp [44]. Moreover, the replication of micro-/nano-structures becomes challenging when the A.R. is greater than 2.0 because of stamp releasing problems due to increased contact area.

Since most superhydrophobic surfaces can be easily contaminated by different types of fat- and oil-based liquids that have much lower surface tension, such as decane (*γ_lv_* = 23.8 mN/m) and octane (*γ_lv_* = 21.6 mN/m) [45], the superhydrophobic applications are limited and challenged in several situations [25]. Instead of using superhydrophobic surfaces, superoleophobic surfaces (organic liquids repellent) and superamphiphobic surfaces (water and oil repellent) are more likely to be used for water-/oil-proof properties in polluted water or greasy environment [25]. Therefore, this study extends to an investigation of the superoleophobicity of the micro-structures.

In this research, the fabrication of high A.R. micro-structures with superhydrophobicity and oleophobicity using R2P NIL process was investigated and demonstrated. The effect of applying a low surface energy resin to replicate micro-structures having A.R. 5.0 onto both polycarbonate (PC) and polyethylene terephthalate (PET) foil substrates on the pattern qualities and pattern fidelity was studied. The effects of the imprinted hierarchical micro-structures on the hydrophobicity, oleophobicity and robustness were investigated as well. Furthermore, the performance of the R2P NIL process in fabricating the water- and oil-repellent properties and robustness of the hierarchical micro-structures has been compared to conventional PDMS micro-structures fabricated by a soft lithography process.

## 2. Materials and Methods

### 2.1. Pattern Design

**Figure 3:**
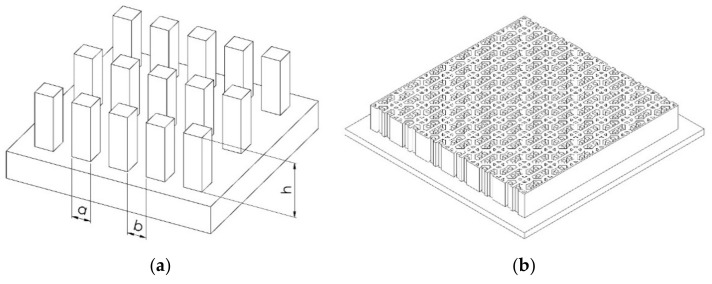
Schematic diagram of the fabricated micro-structures (**a**) square-like pillar (PIL) and (**b**) circular rings with eight stripe supporters (C-RESS) pattern.

In this research, two different micro-structures, including a square-like pillar (PIL) pattern and circular rings with eight stripe supporters (C-RESS) pattern were designed as shown schematically in Figure 3a,b, respectively. These patterns were arranged in a square array. Si master molds of both the PIL and C-RESS micro-structure were fabricated by a conventional photolithography and deep reactive ion etching (DRIE) process [34,36]. In this experiment, the resolution of the lithographic exposure tool called Nikon stepper, model NSR2005i8A (Miyagi Nikon Precision Co., Ltd., Miyaki, Japan) is 0.5 µm. Therefore, the pattern was designed with a size (a) of 0.5 µm, a pattern spacing (b) of 0.5 µm, and a pattern height (h) of 2.5 µm. Hence, the A.R. of the PIL and C-RESS patterns were 5.0.

### 2.2. Silicon Mold Fabrication

**Figure 4:**
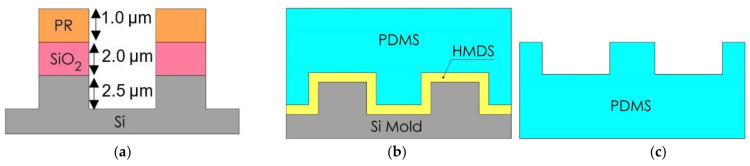
Cross-sectional schematic diagram (not to scale) of (**a**) PR/SiO_2_/Si patterning by conventional photolithography and plasma etching process (**b**) HMDS priming and PDMS casting by soft lithography process, and (**c**) peeled off PDMS sample from the Si master mold.

First, the surface of the 6 inch p-Si (100) wafers was cleaned by standard cleaning (SC-1) process using the automatic wet bench (AWB) machine (STEAG Energy Services GmbH, Essen, North Rhine-Westphalia, Germany) to remove unwanted contaminants [46]. Then, a 2.0 µm-thick silicon dioxide (SiO_2_) layer was deposited onto the Si wafers by means of a low-pressure chemical vapor deposition (LPCVD) process using Samco SVG TMX 2604 (Samco-ucp Ltd., Ruggell Liechtenstein). The O_2_ and H_2_ gas flow rate is 5.0 and 8.5 standard liter per minutes (SLPM), respectively. The oxidation temperature is 1050 °C with a duration time of 13 h. Layers of 1.0 µm-thick Sumitomo PFI-34A photoresist (PR) (Sumitomo chemical advanced technologies, Phoenix, AZ, USA) with either the PIL or C-RESS pattern were fabricated by conventional photolithography process using the Nikon stepper, model NSR2005i8A (Miyagi Nikon Precision Co., Ltd., Miyaki, Japan). The Hg lamp power is 395 mW/cm^2^, exposure time is 920 ms, focus distance is −0.4 µm. After being developed with the Sumitomo SD-W developer (Sumitomo chemical advanced technologies, Phoenix, AZ, USA) for 80 s, the PR patterns were transferred to the SiO_2_ layer by reactive ion etching (RIE) process (CF_4_/CHF_3_/Ar: 10/50/100 sccm, P: 150 mTorr, RF: 800 W/10 min) using the RIE system from Applied Materials, model AMAT P5000 Mark II (Applied Materials, Inc., Santa Clara, CA, USA). Later, the patterns with a height of 2.5 µm were etched into the Si by DRIE from Oxford Instruments, model Plasmalab ICP100 (Oxford Instruments Plasma Technology, Bristol, UK) using Bosch process for 10 cycles, respectively, as shown in Figure 4a. One etching cycle includes a deposition step (C_4_F_8_/SF_6_: 300/5 sccm, ICP/RIE: 2000/0 W, 6 s) and an etching step (C_4_F_8_/SF_6_: 1/200 sccm, ICP/RIE: 2000/15 W, 5 s). After stripping the PR by using oxygen plasma ashing (Ramco RAM-250, Ramco Equipment Corporation, Hillside, NJ, USA) process (RF power 1000 W, O_2_: 900 sccm, gas pressure: 900 mTorr at 80 °C for 1 h), the Si wafers were immersed in piranha acid solution (70 wt% H_2_SO_4_: 30 wt% H_2_O_2_, 4:1 weight ratio) from JT Baker^®^ (Avantor, Phillipsburg, NJ, USA) at 120 °C for 10 min. Then the samples were rinsed with deionized water (DIW) for 15 min and blown dry with pure nitrogen gas. Finally, the remained SiO_2_ hard mask was etched by buffer oxide etching (BOE) process using HF (49% vol): NH_4_F (40% vol) of ratio 1:7 from JT Baker^®^ (Avantor, Phillipsburg, NJ, USA) at 25 °C for 30 min with an etching rate of 900 angstrom per minute.

### 2.3. Soft Lithography Process for PDMS Replication

First, the hydrophilic Si master molds were primed in hexamethyldisilaxane (HMDS) vapor (JT Baker^®^ 9362-09, Avantor, Phillipsburg, NJ, USA) in a desiccator at 25 °C for 48 h to improve their demolding properties as shown in Figure 4b. This prevents getting mold damage from hard PDMS residues in a poor demolding process. The HMDS molecules form a self-assembled monolayer (SAM) onto the Si surface of which the non-polar methyl groups repel water droplets inducing a slight hydrophobicity onto the Si surface (WCA: 60°–80°) with correspondingly good PDMS mold release properties [34,36]. Subsequently, the PDMS mixture (Sylgard 184, curing agent: 10 wt%, Sigma-Aldrich, St. Louis, MO, USA) was poured onto the Si master molds containing either the PIL or C-RESS pattern [34,36]. After curing in a convection oven at 75 °C for 120 min, the PDMS-PIL and PDMS-C-RESS patterns were released from their Si master mold by peeling them by hand, resulting in samples as schematically shown in Figure 4c. A flat PDMS (PDMS-FLT) surface was also fabricated from a non-patterned Si surface as a control sample.

### 2.4. Roll-to-Plate NIL Process for Large-Area Patterning

**Figure 5:**
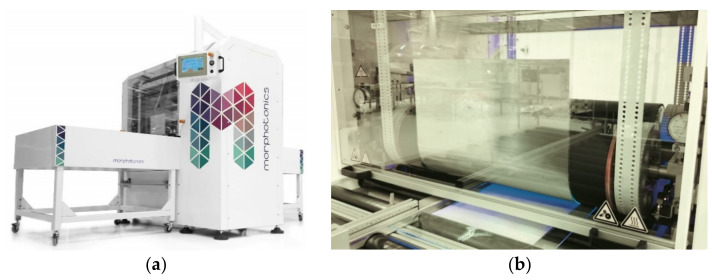
(**a**) Morphotonics’ automated Portis NIL600 tool (Morphotonics, Veldhoven, The Netherlands) and (**b**) set-up of a flexible stamp used for imprinting a substrate coated with UV-curable resin (Morphotonics, Veldhoven, The Netherlands) [47].

**Figure 6:**
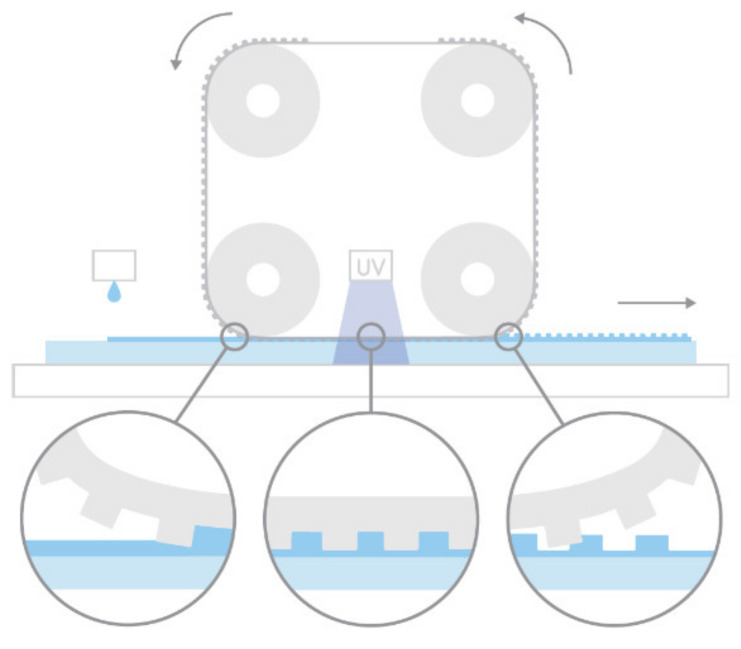
Schematic diagram of Morphotonics’ roll-to-plate ultraviolet nanoimprint lithography (R2P NIL) process (Morphotonics, Veldhoven, The Netherlands) [47].

The R2P NIL process was carried out onto Morphotonics’ automated Portis NIL600 tool (Morphotonics, Veldhoven, The Netherlands) as shown in Figure 5a [47]. Before imprinting, the as-received patterned Si master molds were primed using an in-house releasing agent treatment (Morphotonics, Veldhoven, The Netherlands) with a surface energy of 10–15 mN/m). Subsequently, an in-house transparent flexible stamp is copied from the primed Si master molds in a UV-NIL imprint process. This flexible stamp, containing the inverse PIL or C-RESS micro-structure from the Si master molds and having anti-stick properties, was mounted around the rollers in the Portis NIL600 imprint tool (Morphotonics, Veldhoven, The Netherlands) and an in-house developed UV-curable resin (Morphotonics, Veldhoven, The Netherlands) was dispensed (manually) onto a PC or PET foil substrate which was placed onto a carrier as shown in Figure 5b. Then, the coated substrate was moved to the nip of the imprint rollers and the imprint was performed as follows: as the substrate moves through the nip, the negative structure on the flexible stamp is transferred onto the substrate by laminating the stamp onto the substrate with the UV-curable resin by exerting a uniform line pressure with the imprint roller at a fixed speed. Subsequently, the resin is cured through the transparent stamp by a 365 nm UV-light emitting diode (UV-LED) bar (the UV intensity was set at 0.4 W/cm^2^) after which the stamp is delaminated from the imprinted substrate by the delamination roller. The flexible stamp is then transported back to the start position and can be reused for a next imprint cycle. This can be repeated for more than 1000 times with a single flexible stamp. A schematic of the R2P NIL process is depicted in Figure 6.

For all samples the imprint speed was 0.2 m/min, though it is possible to imprint faster in order to increase throughput (up to 2 m/min was tested for the PIL micro-structure). The gap between imprint roller and substrate was set to −0.75 mm. Both the PIL and C-RESS micro-structures could be imprinted with a broad range of line pressures (2.5–15.0 N/cm), thereby accurately controlling the residual layer thickness. For the C-RESS pattern often the adhesion of either the stamp or the imprint was failing due to the large forces during delamination (due to large surface area), despite using specifically developed in-house resins with low surface free energy (SFE) and good adhesion to both PC and PET. In particular, the two in-house resins that have been used, are: MM1078 (Morphotonics, Veldhoven, The Netherlands), hereafter resin A (viscosity: 149 mPa·s, surface energy: ~15 mN/m, Young’s modulus: 59 MPa) and MM2138C4 (Morphotonics, Veldhoven, The Netherlands), hereafter resin B (viscosity: ~35.3 mPa·s, surface energy: ~10 mN/m, Young’s modulus: ~170 MPa).

### 2.5. Surface Characterizations

After imprinting, the R2P NIL fabricated flexible stamps and samples were characterized by means of a 3D laser scanning confocal microscope (Keyence, VK-X1000 series, Keyence Corporation, Osaka, Japan) in order to inspect the replicated pattern quality and overall defects. Subsequently, the pattern shape and surface topology of both the PDMS and R2P NIL samples were characterized by a field-emission scanning electron microscope (FE-SEM, Hitachi S-4700, Hitachi group, Tokyo, Japan). All samples were coated with 2.0 nm-thick platinum to improve the conductivity of the PDMS and resin patterns.

Droplets of 5.0 μL DIW and ethylene glycol (EG) (Sigma-Aldrich, St. Louis, MO, USA) were dropped onto the patterned surfaces on five different locations of each sample. The measurement was conducted to obtain an average water (*γ_lv_* = 72.1 mN/m) [48] contact angle (WCA) and ethylene glycol (*γ_lv_* = 47.3 mN/m) [48] contact angle (EGCA) by a contact angle goniometer (Ramé-hart instrument Co., Model-400, Succasunna, NJ, USA). The tilt angle of the substrate was 0°. Test conditions are stable in the cleanroom class 1000 with well-controlled environment temperature at 22 ± 1 °C and relative humidity at 45 ± 5%. The contact angle is estimated using the sessile drop technique by measuring the angle between the tangent lines along solid-liquid interface and liquid-vapor interface of the liquid contour.

The advancing contact angle (*θ_adv_*) was measured by adding a volume of liquid to the first drop dynamically for five times (drop volume has increased from 5.0 to 25.0 µL) to the maximum volume of 25.0 µL permitted without increasing the three-phase line. The receding contact angle (*θ_rec_*) was measured by removing a volume of liquid from the drop for 4 times. The drop volume has then decreased from 25.0 to 5.0 µL. When the receding contact angle is subtracted from the advancing contact angle, the result is called the contact angle hysteresis (CAH). The CAH characterizes surface topology and can help quantify contamination, surface chemical heterogeneity, and the effect of surface treatments, surfactants and other solutes [49].

The SFE was measured by a mobile surface analyzer, MSA (KRÜSS GmbH, Hamburg, Germany) and extracted by using Owens, Wendt, Rabel and Kaelble (OWRK) method. In the OWRK model, at least two liquids such as DIW (*γ_lv_* = 72.1 mN/m) and diiodomethane (DI) from Sigma-Aldrich, St. Louis, MO, USA (*γ_lv_* = 50.8 mN/m) with known dispersive ($\gamma_{sv}^{D}$) and polar parts ($\gamma_{lv}^{P}$) of the surface tensions are needed to compute the solid SFE [50]. The total SFE (*γ_sv_*) of the solid is the sum of the two parts as described in Equation (4). The results enable well-founded statements about wettability by aqueous or organic liquids.

(4) $$\;\gamma_{sl}=\;\gamma_{sv}+\gamma_{lv}-2\left(\sqrt{\gamma_{sv}^{D}\gamma_{lv}^{D}}+\sqrt{\gamma_{sv}^{P}\gamma_{lv}^{P}}\right)$$

$\gamma_{sv}^{D}$ and $\gamma_{lv}^{D}$ are the dispersive components and $\gamma_{sv}^{P}$ and $\gamma_{lv}^{P}$ are the polar components of the solid and liquid surface energies, respectively.

Lastly, surface durability was tested by scratching the PDMS and R2P NIL fabricated micro-structures using a glass slide with an applied compressive force while moving the glass slide back and forth 30 times.

## 3. Results and Discussion

### 3.1. PDMS Pattern Qualities and Surface Properties

**Figure 7:**
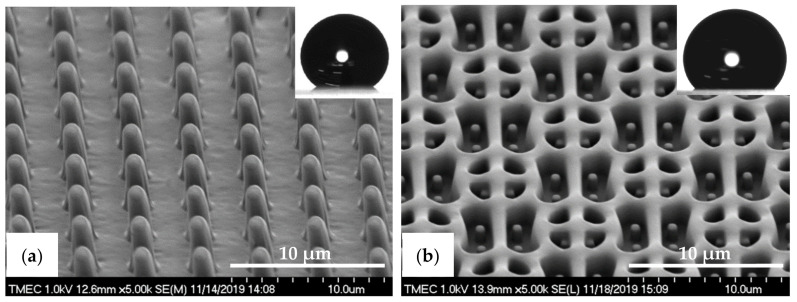
Tilted-view SEM images of the PDMS micro-structures fabricated by soft lithography process before scratch test. (**a**) PDMS-PIL pattern (designed pattern size: diameter: 2.0 µm, height: 2.5 µm) and (**b**) PDMS-C-RESS pattern (designed pattern size: diameter: 1.0 µm, height: 2.5 µm). The insets show water droplet contact angle images on those PDMS surfaces.

In case of the PDMS pattern design, the dimensions of the PIL and C-RESS micro-structures on the Si master molds are (a = 2.0 µm, b = 2.0 µm, h = 2.5 µm) and (a = 1.0 µm, b = 1.5 µm, h = 2.5 µm), respectively. Therefore, the A.R. of these PIL and C-RESS Si micro-structures are 1.25 and 2.50, respectively, which are lower than of the PIL and C-RESS micro-structures on the Si master molds used for the R2P NIL replication processes. Nevertheless, the pattern heights are the same so that a comparison between the replication quality and pattern fidelity of the PDMS soft lithography and R2P NIL fabrication processes can be done to this extent. Note that the feature size was limited to 1.0 µm because here, the Si mold patterning was done using a contact mask aligner (EVG model 620, EV Group Europe & Asia/Pacific GmbH, St. Florian am Inn, Austria). Furthermore, here the Si master molds contain the inverse PIL (i.e., holes) and C-RESS micro-structures because the PDMS soft lithography process consists of only one casting step as opposed to the R2P NIL process used in this study. Figure 7a,b show the SEM images of the PDMS-PIL and PDMS-C-RESS replicated micro-structures before scratch test, respectively. It can be seen that these PDMS patterns were successfully released from the HMDS primed Si master molds. Both PDMS micro-structures were well replicated from the Si molds without defects like pattern clumping, collapsing or breaking off. It is observed that the PDMS patterns have very smooth surfaces without sidewall scallops from the Si mold features, as well as less steep vertical sidewalls. This could possibly have been caused by insufficient curing or a (thermal) reflow effect of the PDMS patterns.

**Figure 8:**
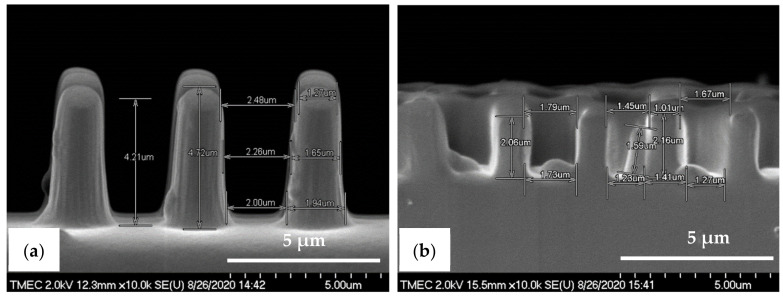
Cross-sectional view SEM images with corresponding measured dimensions of the PDMS micro-structures fabricated by soft lithography process before scratch test. (**a**) PDMS-PIL pattern (designed pattern size: diameter: 2.0 µm, height: 2.5 µm) and (**b**) PDMS-C-RESS pattern (designed pattern size: diameter: 1.0 µm, height: 2.5 µm).

The cross-sectional SEM images in Figure 8a,b show that the pattern width (a) of the PDMS-PIL (a: 1.9 ± 0.02 µm) and PDMS-C-RESS patterns (a: 0.9 ± 0.10 µm) was well controlled resulting in a −5.0% and −10.0% deviation (i.e., shrinkage) from the pattern design, respectively. Note that the vertical sidewalls are less steep so that the width at the top of the pattern is smaller than at the bottom. The pattern heights (h) were measured at 4.8 ± 0.06 µm for the PDMS-PIL and 2.3 ± 0.07 µm for the PDMS-C-RESS pattern resulting in a 92.0% elongation and an 8.0% shrinkage compared to the pattern design, respectively. These numbers result in an A.R. of 2.53 and 2.56 for the PDMS-PIL and PDMS-C-RESS micro-structures, respectively. The differences of the PDMS pattern heights could be explained by a number of factors. Firstly, the pattern depths on the Si master molds could differ due to the micro-loading and the aspect ratio dependent etching (ARDE) effects, which cause the Si etch rate to be dependent on the density and A.R. of the micro-structures, respectively [51,52,53]. Secondly, it could be that the flexible PDMS patterns are stretched during peeling from the Si mold (possibly the case for the PDMS-PIL samples) or have shrunk during curing (possibly the case for the PDMS-C-RESS samples).

In order to investigate the surface wettability behavior, the WCA of the PDMS-PIL and PDMS-C-RESS micro-structures before the scratch test were measured as 145 ± 3.2° and 130 ± 2.4°, respectively. Both patterned PDMS samples did not show any superhydrophobic properties with the WCA greater than 150°. This is due to the still relatively low A.R. of ~2.5 of the designed and subsequently replicated micro-structures. In addition, CAH of the PDMS-PIL and PDMS-C-RESS micro-structures are 1.5° and 10.7°, respectively, showing a better stability of the surface wettability for the PIL pattern.

**Figure 9:**
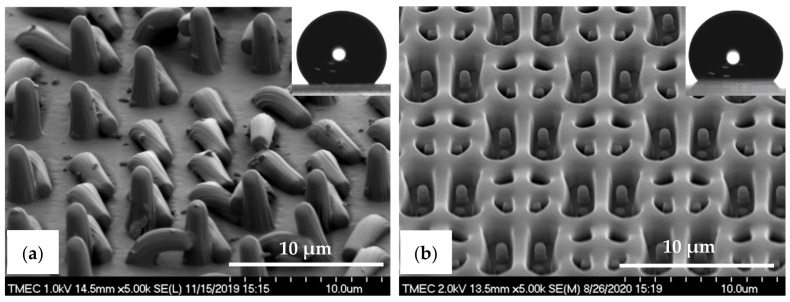
Tilted-view SEM images of the PDMS micro-structures fabricated by soft lithography process after scratch test. (**a**) PDMS-PIL pattern (designed pattern size: diameter: 2.0 µm, height: 2.5 µm) and (**b**) PDMS-C-RESS pattern (designed pattern size: diameter: 1.0 µm, height: 2.5 µm). The insets show water droplet contact angle images on those PDMS surfaces.

Next, the robustness of the PDMS replicated patterns was studied by means of a scratch test. After the scratch test, the PDMS-PIL pattern was mostly found collapsed due to the applied external forces, as shown in Figure 9a. The pattern collapsing was caused by the poor mechanical strength of the PDMS. Furthermore, pattern mating and clumping were also observed after the scratch tests. These defects are due to the fact that the Van Der Waals force between the adjacent patterns is larger than pulling force or recovery force. Moreover, the electrostatic discharge (ESD) induced during the scratch test can generate an adhesion force between the pillars. In contrast, the PDMS-C-RESS pattern did not collapse after the scratch tests and did not exhibit other defects or damage, as shown in Figure 9b. This is due to the robust design of the C-RESS micro-structure pattern.

After scratch tests, the WCA of the PDMS-PIL sample was decreased to 135.9 ± 9.5° due to the induced defects, while the WCA of the PDMS-C-RESS pattern stayed quite the same at 130.0 ± 4.9°, respectively. It was also found that the standard deviation (S.D.) of the WCA of the PDMS-PIL pattern has increased after scratch tests. Moreover, the CAH of the PDMS-PIL pattern has increased significantly to 21.4°. This is due to the variation of the surface roughness across the PDMS-PIL surface due to the pattern collapsing, mating, and clumping. In contrast, the S.D. and CAH (11.5°) of the PDMS-C-RESS patterns did not really increase because the C-RESS micro-structure is robust enough to maintain the pattern shape and surface roughness after the scratch test. Based on the results, the C-RESS pattern is considered attractive for antifouling applications because of its robustness and already quite high hydrophobicity. However, the pattern width and spacing should be further reduced and the pattern height should be further increased to reach an A.R. of 5.0, which is minimally needed in order to obtain superhydrophobicity as well as superoleophobic properties.

### 3.2. R2P NIL Pattern Qualities and Surface Properties

**Figure 10:**
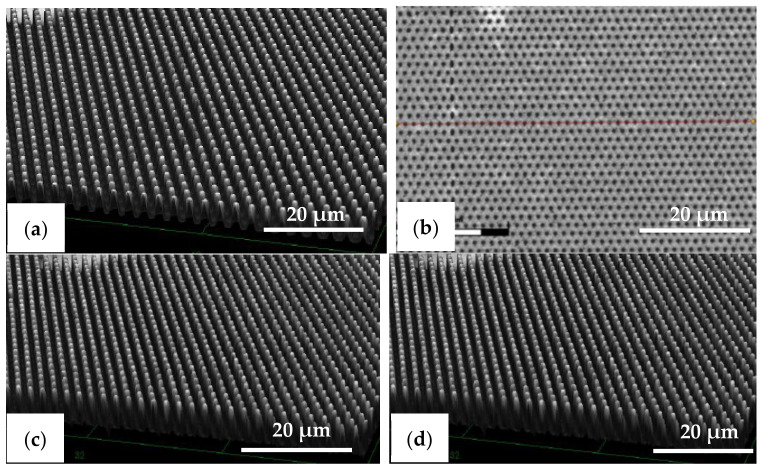
Laser confocal microscopy images of the pillar micro-structure (designed pattern size: diameter: 0.5 µm, height: 2.5 µm) on the (**a**) Si master mold before in-house releasing agent treatment, (**b**) R2P NIL flexible stamp (holes, top-view), (**c**) imprint on PC foil with resin A (PIL-A), and (**d**) imprint on PC foil with resin B (PIL-B).

**Figure 11:**
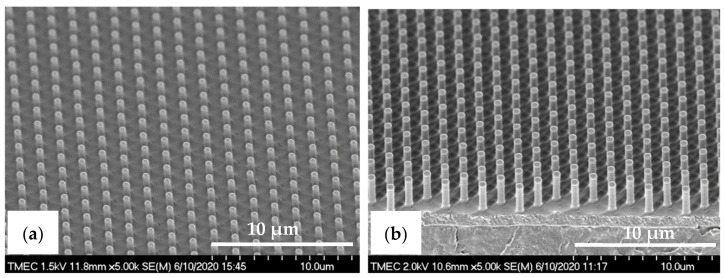
Tilted-view SEM images of the pillar micro-structure (designed pattern size: diameter: 0.5 µm, height: 2.5 µm) replicated by R2P NIL process (**a**) imprint on PC foil with resin A (PIL-A) and (**b**) imprint on PC foil with resin B (PIL-B).

**Figure 12:**
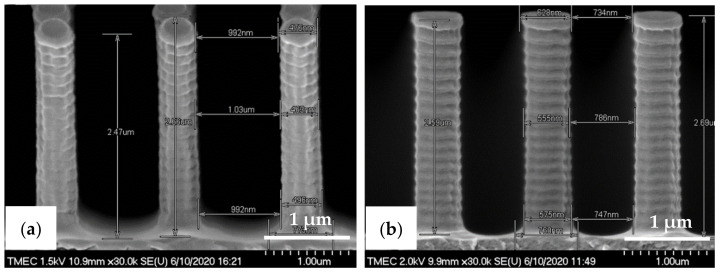
Cross-sectional view SEM images with corresponding measured dimensions of the pillar micro-structure (designed pattern size: diameter: 0.5 µm, height: 2.5 µm) replicated by R2P NIL process (**a**) imprint on PC foil with resin A (PIL-A) and (**b**) imprint on PC foil with resin B (PIL-B).

The R2P NIL imprints have been made using another Si master mold. The laser confocal microscopy image of the pillar (PIL) micro-structure on the Si mold in Figure 10a shows that the DRIE process at TMEC was well-controlled resulting in a high-quality pattern with no defects or contamination. This confocal microscope gives a general quality indication. Due to the steep angles the confocal microscope cannot determine the structure dimensions (and therefore not replication fidelity) accurately. SEM characterization and step profilometer measurement confirmed that the etch depth of the Si micro-structures was found uniform across a six-inch Si wafer with only 2.0% deviation from the pattern design (data not shown). A precise and uniform flexible stamp (intermediate resin mold) containing the inverse polarity of the PIL pattern (i.e., holes) was well fabricated from the in-house releasing agent treated Si master mold, as shown in Figure 10b. Using the R2P NIL process, the pillar micro-structure from the Si mold (A.R. of 5.0) was well replicated onto both PC and PET foil substrates with both resin A (PIL-A) and resin B (PIL-B), as shown in Figure 10c,d. According to the laser confocal microscopy images, no pattern clumping, collapsing or breaking off of the micro-pillars was found on the Si master mold and the imprinted samples PIL-A and PIL-B, as shown in Figure 10a,c,d, respectively. Moreover, no air bubble defects were present on the samples after imprinting. These results are further corroborated by SEM analysis of the samples, which gives a more detailed view of the replicated pattern quality and allows for an accurate determination of the replication fidelity. This is important for a good evaluation of the antifouling behavior of the imprinted micro-structures. The SEM images of the pillar micro-structures imprinted with resin A (PIL-A) and resin B (PIL-B) before scratch test are shown in Figure 11a,b, respectively. It can be seen that, with the aid of Morphotonics’ in-house Si mold releasing agent and due to the low SFE of the used resins, the micro-pillars were well replicated from the Si master mold and subsequently from the flexible stamp within the R2P NIL process. Again, no pattern clumping, collapsing or breaking down was found as well as no air bubble defects. Furthermore, in Figure 12a,b, the cross-sectional SEM images show that the pattern width (a) of the PIL-A (a: 458.3 ± 20 nm) and PIL-B patterns (a: 586.0 ± 37 nm) was different resulting in a −8.3% and +17.2% deviation from the original design, respectively, despite having experienced the same R2P NIL fabrication process.

The almost vertical sidewalls and rough surfaces with sidewall scallops from the Si master mold were also observed on the PIL-A and PIL-B patterns, while they were absent on the PDMS samples. This underlines the high patterning resolution of the R2P NIL process. The pattern height (h) was measured at 2.57 ± 0.13 µm and 2.64 ± 0.07 µm, respectively. Calculation of the imprinted pattern height deviation from the original design gives a 2.8 and 5.6% elongation resulting in an A.R. of 5.6 and 4.5 for the PIL-A and PIL-B micro-structures, respectively. These differences are likely attributed to the discrepancy between the original design and the actual Si mold pattern heights and to the different resin properties which result in different polymerization shrinkage values upon UV curing and different flexibilities during delamination, amongst others. Here, due to the large surface area the forces during delamination are such high that the cured micro-pillars could have been stretched out more than they have shrunk.

**Figure 13:**
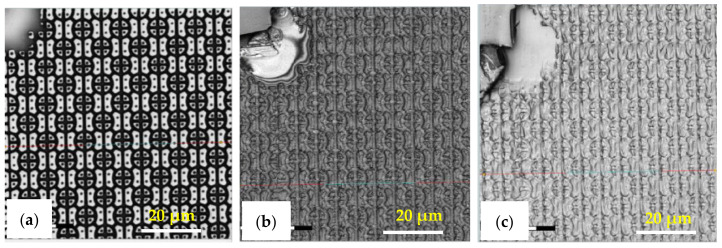
Top-view laser confocal microscopy images of the C-RESS micro-structure (pattern design: size: 1.5 µm, space: 0.5 µm, height: 2.5 µm) on the (**a**) Si master mold before in-house releasing agent treatment (i.e., with HMDS vapor priming), (**b**) imprint on PET foil with resin A (C-RESS-A), and (**c**) imprint on PET foil with resin B (C-RESS-B). Note that the artefact seen in the top left corner is part of the design on the Si master mold and is therefore copied onto the samples.

Besides replicating the micro-pillar pattern, also fabrication of the more robust C-RESS micro-structure with the R2P NIL process was investigated. Without Morphotonics’ in-housing Si mold releasing agent treatment (i.e., with the HMDS vapor primed Si wafer) the C-RESS structure was not successfully replicated. The laser confocal microscopy images in Figure 13 show that the patterns are completely delaminated/broken off from the substrate, as can been seen from the comparison with the Si master mold pattern. This is caused by the high forces during delamination of the flexible stamp from the cured patterns, which is due to the large surface area of the C-RESS micro-structure and the relatively high SFE of the HMDS primed Si master mold and, subsequently, of the flexible stamp, despite the use of the low SFE resins.

**Figure 14:**
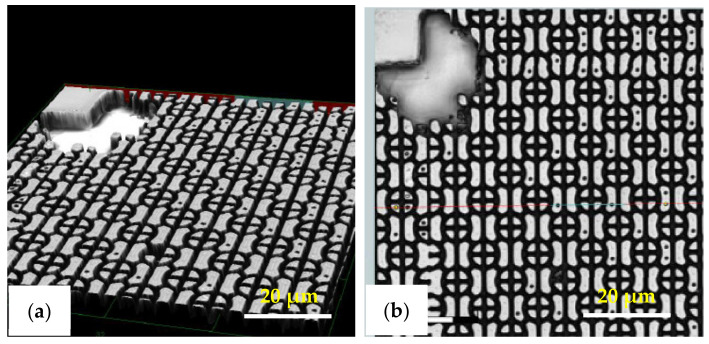
Laser confocal microscopy images of the C-RESS micro-structure (pattern design: size: 1.5 µm, space: 0.5 µm, height: 2.5 µm) on the (**a**) imprint on PC foil with resin A (C-RESS-A), and (**b**) imprint on PC foil with resin B (C-RESS-B).

With the aid of Morphotonics’ in-house Si mold releasing agent, however, the replication of the C-RESS pattern was significantly improved though still challenging and several defects were observed. Firstly, as shown in Figure 14, it is observed on the imprinted C-RESS-A and C-RESS-B samples that many of the small holes are filled instead of well-replicated. This could be explained by the incomplete filling of the holes in the Si master mold during fabrication of the flexible stamp or by the breaking off of the pillars on the flexible stamp during delamination. Secondly, it can be seen that sometimes also other parts of the C-RESS pattern can be broken off, again indicating the high forces that are present during delamination. Nevertheless, these results show that the choice of Si mold releasing agent in conjunction with the used resins and flexible stamp materials and the imprint settings is key in achieving a good replication of the high A.R. C-RESS micro-structure.

**Figure 15:**
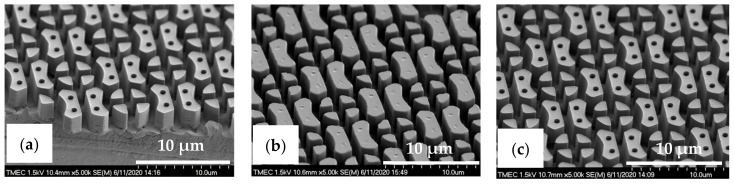
Tilted-view SEM images of C-RESS micro-structure (pattern design: size: 1.5 µm, space: 0.5 µm, height: 2.5 µm) replicated by R2P NIL process (**a**) imprint on PC foil with resin A (C-RESS-A), and (**b**,**c**) imprints on PC foil with resin B (C-RESS-B).

**Figure 16:**
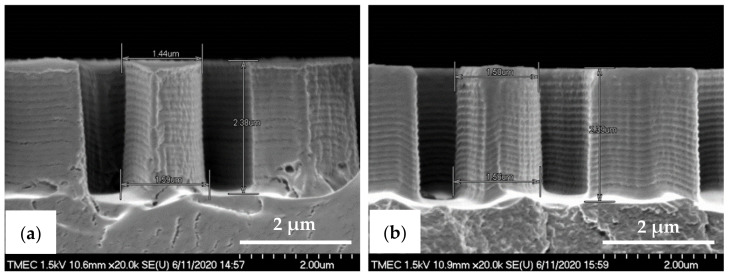
Cross-sectional view SEM images with corresponding measured dimensions of the C-RESS micro-structure (pattern design: size: 1.5 µm, space: 0.5 µm, height: 2.5 µm) replicated by R2P NIL process (**a**) imprint on PC foil with resin A (C-RESS-A) and (**b**) imprint on PC foil with resin B (C-RESS-B).

Here, the intermediate flexible stamp (not shown) used was made from the Si master mold after the in-house releasing agent treatment. Note that the artefact seen in the top left corner is part of the design on the Si master mold (see Figure 13a) and is, therefore, copied onto the samples. Detailed SEM imaging of the C-RESS-A and the C-RESS-B micro-structures before the scratch test further corroborate the abovementioned results and are shown in Figure 15a–c, respectively. Although the first two SEM images seem to presume that the replications were perfect and without defects, they are very local recordings of the patterned area. Moreover, a third SEM image shows indeed that many of the filled holes seem to be due to the breaking off of the pillars on the flexible stamp during delamination. Furthermore, no pattern mating, clumping or collapsing of the robust C-RESS micro-structure was found on the imprints as well as no air bubble defects. The cross-sectional SEM images in Figure 16a,b show that the pattern width (a) of the C-RESS-A (a: 1.52 ± 0.11 µm) and PIL-B patterns (a: 1.53 ± 0.04 µm) was practically the same and only deviating 1.3% and 2.0% from the original design, respectively.

Similar to the pillar micro-structure the C-RESS-A and C-RESS-B replicated patterns also exhibit the rough surfaces with sidewall scallops due to the Si master mold fabrication (DRIE process), again showing the high patterning resolution of the R2P NIL process. The pattern height (h) was measured at 2.38 ± 0.04 µm and 2.39 ± 0.06 µm, respectively. This results in a pattern height shrinkage of 4.8% and 4.4% compared to the original design. Since the pattern fidelity values for both the C-RESS-A and C-RESS-B samples are so close to each other, it may be concluded that the two resins exhibit similar shrinkage upon UV curing (for this particular micro-structure). It is likely the challenging design of the C-RESS pattern that limits a flawless replication.

The difference between the replicated PIL and the C-RESS pattern heights (but also part of the observed deviations from the pattern design) can be explained by a different pattern depth on the Si master molds due to the earlier mentioned micro-loading and ARDE effects, which cause the Si etch rate to be dependent on the density and A.R. of the micro-structures [51,52,53]. Taking this into consideration, it may be that the actual pattern height fidelity of the C-RESS micro-structure replications are possibly even better and that the A.R. could be close to 5.0.

In order to examine the surface wetting behavior, each pattern was fabricated multiple times to investigate the reproducibility of the R2P NIL process by measuring sample-to-sample deviations. Therefore, the micro-structure replicated with resin A was labeled with sample identification numbers (sample ID) as PIL-A-1, PIL-A-2, C-RESS-A-1, and C-RESS-A-2 and the micro-structure replicated with resin B as PIL-B-1, PIL-B-2, C-RESS-B-1, and C-RESS-B-2. The WCA of the PIL-A, PIL-B, C-RESS-A, and C-RESS-B samples before the scratch test was measured as shown in Table 1 and the EGCA was measured as shown in Table 2. In addition, the advancing and receding WCA and EGCA are presented in Table 1 and Table 2 as well as the respective CAH.

**Figure 17:**
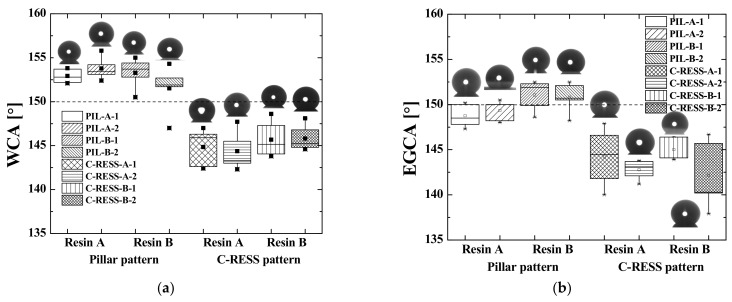
Effects of micro-structure pattern and resin type on the contact angle of (**a**) water (WCA) and (**b**) ethylene glycol (EGCA) of the imprinted samples.

**Figure 18:**
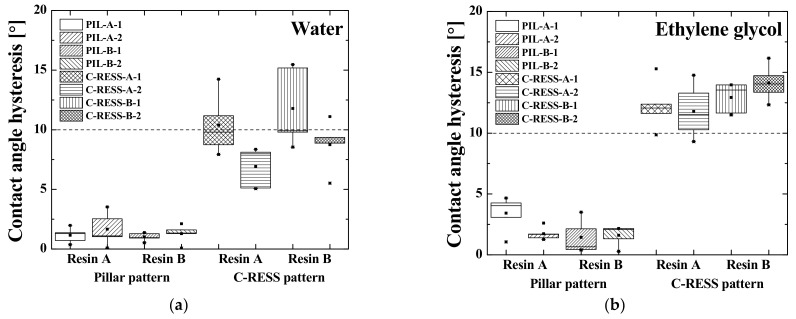
Effects of micro-structure pattern and resin type on the contact angle hysteresis (CAH) of (**a**) water droplets and (**b**) ethylene glycol droplets dispensed on the imprinted samples.

Furthermore, the WCA, the EGCA and their corresponding droplet shapes on the different micro-structures before scratch test are shown in Figure 17a,b, respectively. From a first glance, it can be concluded that there is no significant difference of the surface wettability between the patterns replicated with resin A and resin B. This shows that the SFE of the cured imprint resins does not necessarily has to be very low, because the A.R. of the micro-structure patterns themselves (~5.0) already induce a large portion of the surface (super) hydrophobicity. In order to make a strong conclusion about this, the micro-structures should be replicated using higher SFE resins as well. These higher SFE will have the difficulty that, most probably, the imprint will fail due to stamp sticking problems during delamination. Figure 18a,b show the CAH of the water and the ethylene glycol (EG) droplets on the various replicated micro-structure surfaces. The water CAH of the PIL and the C-RESS micro-structures are in the range of 1.0 ± 0.3° to 1.7 ± 1.3° and 6.9 ± 1.7° to 11.8 ± 2.0°, respectively. The EG CAH of the PIL and the C-RESS micro-structures are in the range of 1.4 ± 1.3° to 3.4 ± 1.4° and 11.8 ± 2.3° to 15.3 ± 8.5°.

From this, it can be concluded that the surface wettability of the PIL micro-structure is more stable than that of the C-RESS micro-structure because of the lower value of CAH. This can be explained by the better imprint quality and fidelity of the PIL micro-structure than compared to the C-RESS micro-structure. In addition, the PIL-A and the PIL-B micro-structures show superhydrophobicity and superoleophobicity because the WCA and the EGCA were greater than 150° and the value of the water CAH and EG CAH was lower than 10°. In contrast, the C-RESS-A and the C-RESS-B micro-structures show only hydrophobicity and oleophobicity, although the WCA and EGCA values are not that much lower than of the PIL-A and PIL-B samples. This is likely due to the fact that the C-RESS pattern was replicated less well by the R2P NIL process and with more artefacts (such as the filled holes) compared to the PIL pattern. Nevertheless, if the pattern fidelity of the C-RESS micro-structure can be improved, the surface wettability may become superhydrophobic and superoleophobic.

Furthermore, the hydrophobicity (and oleophobicity) of both the PIL and C-RESS patterns replicated by the R2P NIL process is higher than that of the PDMS samples fabricated by the (conventional) soft lithography process. This is mostly due to the higher A.R. of the patterns on the R2P NIL samples (~5.0) compared to those on the PDMS samples (~2.5). In addition, there could also be differences in the surface wettability due to the different properties of the used materials. However, since the SFE of the used resins and of PDMS are quite similar, these differences are expected to be very minor. Nevertheless, it could be that the better replication quality of the R2P NIL samples (as opposed to the smoothed PDMS patterns) results in the very high WCA and EGCA values in combination with the higher A.R. Moreover, the R2P NIL patterns showed lower water CAH and EG CAH compared to the PDMS samples. This means that the R2P NIL process has a higher patterning resolution and higher pattern fidelity with better uniformity across the sample surfaces compared to the conventional soft lithography process.

### 3.3. Effects of Micro-Structure Pattern and Resin Types on the Surface Energy

Table 3 shows the WCA and diiodomethane contact angle (DICA) of the PIL-A, PIL-B, C-RESS-A, and C-RESS-B imprinted patterns. Note that the data were obtained using the mobile surface analyzer (MSA) system (KRÜSS GmbH, Hamburg, Germany), which is a different contact angle measurement tool than what has been used in the previous section. Therefore, these contact angle values are slightly different.

**Figure 19:**
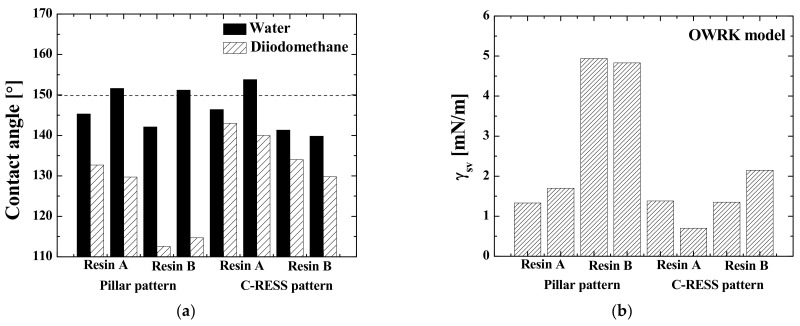
Effects of micro-structure pattern and resin type on the surface properties (**a**) water and diiodomethane contact angle and (**b**) surface free energy of the imprinted micro-structures.

The WCA values of all samples were higher than their DICA values, as shown in Figure 19a. This is an effect of the lower value of the water surface tension (*γ_lv_* = 72.1 mN/m) compared to that of diiodomethane from Sigma-Aldrich, St. Louis, MO, USA (*γ_lv_* = 50.8 mN/m). The values of the WCA and DICA were used to calculate the SFE (*γ_sv_*) of the imprinted samples using the OWRK model. It was found that the *γ_sv_* of the PIL-A and PIL-B patterns before the scratch test was in the range of 1.33 ± 0.4 to 1.70 ± 0.4 mN/m and 4.83 ± 1.9 to 4.94 ± 1.7 mN/m, respectively, and that of the C-RESS-A and C-RESS-B patterns in the range of 0.70 ± 0.2 to 1.38 ± 2.5 mN/m and 1.35 ± 0.6 to 2.15 ± 1.1 mN/m, respectively. Figure 19b shows that the *γ_sv_* of the micro-structures imprinted with resin A is lower than that of the micro-structures imprinted with resin B, even though the SFE of a flat sample of resin A (*γ_sv_*~15 mN/m) is higher than that of a flat sample of resin B (*γ_sv_*~10 mN/m). This difference is especially pronounced for the PIL pattern. This effect is not very well understood yet, since it contrasts to the results in the previous section. However, it is possible that there could be variations in replication quality and pattern fidelity between the samples. Moreover, since resin B is still quite experimental, it could be that its properties change over time or are inhomogeneous across multiple samples, thereby causing this discrepancy. More research should be performed in order to fully understand this.

### 3.4. Robustness of the R2P NIL Imprinted Micro-Structures after Scratch Test

**Figure 20:**
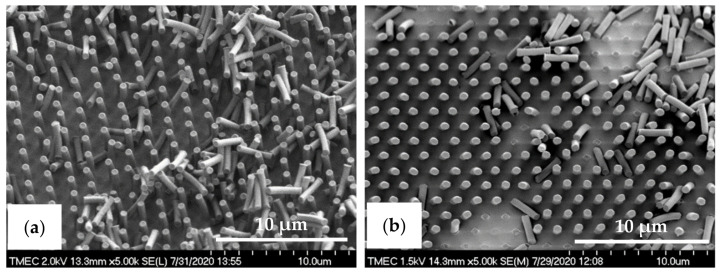
Top-view SEM images of the R2P NIL replicated pillar micro-structure (designed pattern size: diameter: 0.5 µm, height: 2.5 µm) after scratch test (**a**) imprint on PC foil with resin A (PIL-A) and (**b**) imprint on PC foil with resin B (PIL-B).

**Figure 21:**
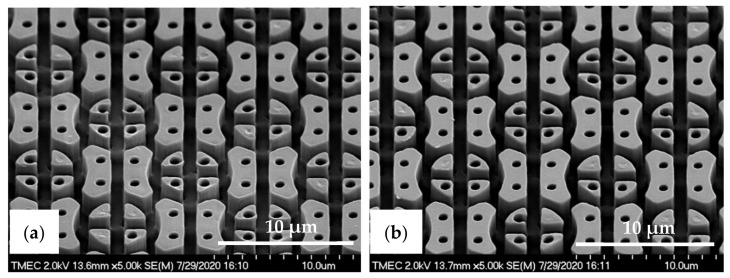
Tilted-view SEM images of the R2P NIL replicated C-RESS micro-structure (pattern design: size: 1.5 µm, space: 0.5 µm, height: 2.5 µm) after scratch test (**a**) imprint on PC foil with resin A (C-RESS-A) and (**b**) imprint on PC foil with resin B (C-RESS-B).

After performing scratch tests, SEM analysis was carried out again and the WCA and EGCA were remeasured in order to investigate the robustness of the R2P NIL replicated micro-structures. The PIL-A and PIL-B micro-structures were mostly found collapsed, twisted or even broken off by the applied external forces, as is shown in Figure 20. Besides pillar collapse and the removal of pillars also pattern mating and clumping were observed. This is due to the fact that the Van Der Waals force between the pillars is larger than the pulling force or recovery force [54]. Moreover, the ESD induced during the scratch test can generate an adhesion force between the pillars. The collapsing or breaking off of the micro-pillars was caused by their poor (inherent) mechanical strength and the low Young’s modulus of the used resins. Although the latter is higher for the used R2P NIL resins than compared to PDMS in the reference soft lithography fabrication process, it is still insufficient for use as a robust anti-biofouling coating. To this end, the C-RESS micro-structure pattern was designed. After the scratch tests, both the C-RESS-A and C-RESS-B patterns remained intact, as shown in Figure 21.

For both samples, there is no pattern collapsing, twisting or breaking off observed. This result shows that the mechanical strength of the used imprint resins is sufficiently high when used in combination with the robustly designed C-RESS micro-structure pattern in order to withstand the scratch test. After the scratch tests, the WCA and EGCA of the PIL-A, PIL-B, C-RESS-A, and C-RESS-B imprinted micro-structures were found to be reduced to (143.3 ± 2.0° and 137.3 ± 4.0°), (127.4 ± 9.3° and 109.1 ± 12.1°), (135.8 ± 5.6° and 135.5 ± 8.2°), and (138.8 ± 5.0° and 121.9 ± 9.4°), respectively. As shown in Table 4, it was also found that the S.D. of the WCA and EGCA of the PIL-A and PIL-B micro-structures significantly increased after the scratch tests. This can be explained by the variation of the surface roughness across the sample surface due to the collapsing, clumping and breaking off of the pillars. Surprisingly, the S.D. of the WCA and EGCA of the C-RESS-A and C-RESS-B patterns increased as well after the scratch test, despite the C-RESS micro-structure being robust enough to preserve its original pattern structures. Furthermore, the WCA and EGCA of the PIL-B sample were found to be significantly lower than those of the PIL-A and even the C-RESS-A and C-RESS-B imprints after the scratch tests. Additionally, the CAH values of the PIL-B sample were much higher after the scratch test than before. Therefore, it could be that the PIL-B imprinted pattern also has a higher degree of chemical degradation of the surface besides the mechanical defects, although this is difficult to conclude from this small sample set.

**Figure 22:**
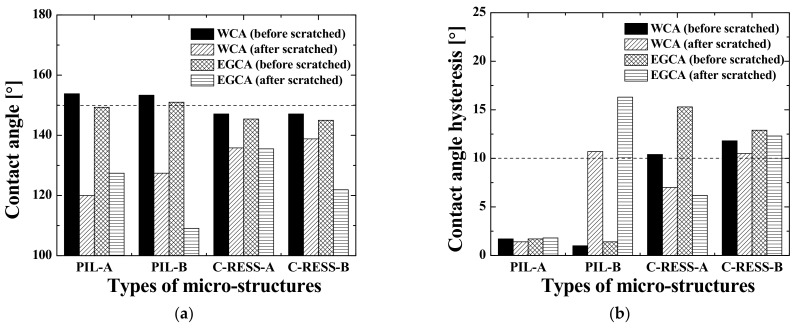
Comparison of the surface wetting properties of the PIL and C-RESS replicated micro-structures before and after scratch tests (**a**) water and ethylene glycol contact angle (WCA and EGCA) and (**b**) contact angle hysteresis (CAH).

The decrease in the WCA and EGCA of the C-RESS-A and C-RESS-B patterns might be related to the chemical degradation of the surface after the scratch tests. For example, the resin surface properties, including surface charge, surface free energy (wettability), and nano-scale surface roughness could have been altered after scratching [55]. Compared to the PIL micro-structure, the replicated C-RESS patterns have a higher (mechanical) durability resulting in a smaller decrease in the WCA and EGCA after the scratch test, as shown in Figure 22.

There was no significant difference of the WCA and EGCA between the C-RESS-A and C-RESS-B imprints after the scratch test, even though the SFE of resin A (*γ_s_*~15 mN/m) is higher than that of resin B (*γ_s_*~10 mN/m). However, the CAH of the water and EG droplets on the C-RESS-B sample was found to be larger than those on the C-RESS-A sample, which could point to additional differences in the surface quality between resin A and B after the scratch test. Nevertheless, if the R2P NIL replication quality of the C-RESS micro-structure can be improved, the initial surface wettability will be better and the small decrease in the WCA and EGCA after scratch test could be acceptable for robust large-area antifouling application.

In summary, based on the above-mentioned results, the C-RESS micro-structure fabricated by R2P NIL using resin A is considered attractive for robust large-area antifouling purposes because of three main benefits. Firstly, although the high A.R. C-RESS micro-structure was challenging to imprint, the pattern fidelity was not too bad (<5% shrinkage compared to the pattern design) so that the A.R. remained close to 5.0. These imprints already result in a hydro- and oleophobicity with the WCA and EGCA of up to 144.6 ± 2.9° and 144.5 ± 2.7° before scratch test, respectively. These values could even be higher if the replication quality can be improved by reducing the amount of filled holes. Moreover, Morphotonics can scale up this pattern from the 6 inch Si wafers to square-meter sized areas, which can then be imprinted more than a thousand times using a single flexible stamp, making the R2P NIL process highly cost-effective.

Secondly, the unique surface topology of the C-RESS pattern may create an air cushion layer even when the surface did not qualify as a superhydrophobic and superoleophobic surface in the Cassie-Baxter regime (WCA and EGCA > 150°). This air cushion layer changes the solid–liquid interface of the C-RESS micro-structure to a combination of solid-liquid and air-liquid interfaces [25,56]. Therefore, the contact area between the micro-organisms and the C-RESS pattern is reduced. Consequently, the surface adhesion force of the micro-organisms decreases and the interaction strength is also reduced [57]. It was also reported that the high A.R. C-RESS pattern with hydrophobic and oleophobic properties reduced the adhesion strength of the adhesive layer, called extracellular polymeric substance (EPS), which can reduce the formation of a biofilm and subsequently suppresses the accumulation of micro-organisms on the material surface [36].

Thirdly, due to its robustness, the imprinted C-RESS pattern can maintain its pattern shape during the scratch tests and, thereby, largely its hydrophobicity and oleophobicity. It can be concluded that the robust C-RESS micro-structure fabricated with a resin having properties like resin A by using the R2P NIL process available at Morphotonics is expected to be one of the promising robust antifouling technologies for large-area medical and marine applications.

Nevertheless, the long-term mechanical durability and chemical stability of the C-RESS micro-structure and its hydro- and oleophobicity are still among the most important challenges. It has been investigated that the dewetting properties and the liquid contact angles of the (conventional) micro-structures can change over time in laboratory environments already and can severely degrade in realistic application scenarios under various working conditions (e.g., low or high alkalinity, salinity, ions, photodegradation, and high temperatures) [58,59]. This might be due to the degradation of the micro-structure patterns and the alteration of the chemical composition of the top surface, which increase the material surface energy and decrease the hydro- and oleophobic properties [58]. However, our previous report showed that the unique surface topology and durability of the PDMS-C-RESS micro-structure can maintain the level of the surface roughness and its hydrophobicity during a field test in seawater environment for 5 months [36]. In future work, such tests should also be conducted for the R2P NIL fabricated C-RESS patterns in order to investigate the performance and long-term mechanical and chemical stability in the field of the samples made by this method.

## 4. Conclusions

The robustness of micro- or nano-structures with a high aspect ratio (A.R.) and the scalability of the large-area patterning and mass production of it, are one of the most critical issues to produce cost-effective antifouling surfaces with both superhydrophobic and superoleophobic properties. In this research, a micro-pillar pattern of A.R. 5.0 was successfully replicated by Morphotonics’ R2P NIL process on their automated Portis NIL600 system using in-house UV-curable resins with low SFE onto PC and PET foil substrates, as an alternative fabrication method for the conventional PDMS soft lithography process. The imprinted micro-pillar (PIL) pattern of A.R. 5.0 showed excellent replication quality without defects and good pattern fidelity (<6% pattern height deviation) which resulted in superhydrophobicity and oleophobicity (WCA above 150°). Furthermore, the well-designed robust circular rings with eight stripe supporters (C-RESS) pattern of A.R. 5.0 was replicated with the same process and materials. The replication quality of this challenging C-RESS imprint pattern was not perfect yet showing defects like filled holes and delaminated features due to large delamination forces. Nevertheless, the imprinted C-RESS micro-structure exhibited a good pattern fidelity (<5% pattern height deviation), already resulting in high hydro- and oleophobicity (WCA above 145°). In addition, the imprinted C-RESS pattern has a good durability during scratch tests, thereby maintaining its pattern shape and largely also its hydro- and oleophobicity. The R2P NIL results have been compared to PDMS samples fabricated by a (conventional) soft lithography process. These samples have a lower WCA due to lower A.R. and lower replication quality.

Therefore, the R2P NIL process available at Morphotonics is expected to be a promising patterning process to fabricate large-scale C-RESS micro-structure patterned substrates with antifouling properties in practical utilization for medical and marine applications.

## Figures and Tables

**Table 1:** Measured water contact angle (WCA) and its contact angle hysteresis (CAH) of the PIL and C-RESS micro-structures replicated with resin A and resin B on PC foil. Note that for each sample the WCA was measured on five different locations across the sample.

Scheme	WCA [°]	WCA_adv_ [°]	WCA_rec_ [°]	CAH [°]
PIL-A-1	152.9 ± 0.8	151.4 ± 2.2	150.2 ± 2.2	1.1 ± 0.6
PIL-A-2	153.8 ± 1.3	151.8 ± 1.4	148.5 ± 1.2	1.7 ± 1.3
PIL-B-1	153.3 ± 1.8	152.5 ± 0.5	151.5 ± 0.4	1.0 ± 0.3
PIL-B-2	151.6 ± 2.5	150.7 ± 0.6	149.5 ± 1.0	1.3 ± 0.8
C-RESS-A-1	147.1 ± 3.6	141.8 ± 2.4	130.9 ± 3.6	10.4 ± 2.5
C-RESS-A-2	144.6 ± 2.9	137.9 ± 1.4	131.5 ± 1.4	6.9 ± 1.7
C-RESS-B-1	147.1 ± 2.9	141.8 ± 3.3	130.0 ± 3.8	11.8 ± 2.0
C-RESS-B-2	144.6 ± 2.1	137.9 ± 1.8	129.2 ± 1.5	8.8 ± 2.7

**Table 2:** Measured ethylene glycol contact angle (EGCA) and its contact angle hysteresis (CAH) of the PIL and C-RESS micro-structures replicated with resin A and resin B on PC foil. Note that for each sample the EGCA was measured on five different locations across the sample.

Sample ID	EGCA [°]	EGCA_adv_ [°]	EGCA_rec_ [°]	CAH [°]
PIL-A-1	148.8 ± 1.3	150.7 ± 2.1	147.3 ± 1.2	3.4 ± 1.4
PIL-A-2	149.3 ± 1.2	149.7± 0.9	147.9 ± 0.9	1.7 ± 0.5
PIL-B-1	151.0 ± 1.7	150.2 ± 2.0	148.9 ± 0.6	1.4 ± 1.3
PIL-B-2	150.8 ± 1.7	150.9 ± 1.0	149.3 ± 0.7	1.6 ± 0.8
C-RESS-A-1	145.4 ± 3.9	144.1 ± 4.1	128.8 ± 5.9	15.3 ± 8.5
C-RESS-A-2	144.5 ± 2.7	137.4 ± 2.6	125.4 ± 2.3	11.8 ± 2.3
C-RESS-B-1	145.0 ± 1.3	140.4 ± 1.7	127.4 ± 2.0	12.9 ± 1.2
C-RESS-B-2	142.2 ± 3.8	139.2 ± 3.1	125.1 ± 2.4	14.1 ± 1.4

**Table 3:** Calculated surface free energy (SFE) values of the PIL and C-RESS micro-structures replicated with two different resins on PC foil, as measured by using water and diiodomethane.

Sample ID	WCA [°]	DICA [°]	$\gamma_{sv}^{D}\;[\mathrm{mN}/m]$	$\gamma_{sv}^{P}\;[\mathrm{mN}/m]$	$\gamma_{sv}\;[\mathrm{mN}/m]$
PIL-A-1	145.3 ± 5.9	132.7 ± 2.9	1.31 ± 0.3	0.02 ± 0.1	1.33 ± 0.4
PIL-A-2	151.6 ± 2.1	129.7 ± 2.9	1.65 ± 0.4	0.05 ± 0.1	1.70 ± 0.4
PIL-B-1	142.1 ± 6.2	112.6 ± 5.5	4.81 ± 1.4	0.13 ± 0.3	4.94 ± 1.7
PIL-B-2	151.2 ± 1.5	114.7 ± 6.3	4.30 ± 1.5	0.53 ± 0.4	4.83 ± 1.9
C-RESS-A-1	146.4 ± 3.1	143.0 ± 1.3	0.90 ± 1.1	0.46 ± 1.3	1.38 ± 2.5
C-RESS-A-2	153.8 ± 1.5	140.0 ± 2.8	0.70 ± 0.2	0	0.70 ± 0.2
C-RESS-B-1	141.3 ± 3.9	134.0 ± 3.9	1.19 ± 3.4	0.17 ± 0.2	1.35 ± 0.6
C-RESS-B-2	139.8 ± 5.8	129.8 ± 3.4	1.65 ± 0.4	0.50 ± 0.7	2.15 ± 1.1

**Table 4:** WCA and EGCA and its contact angle hysteresis (CAH) of PIL and C-RESS micro-structure with resin A and resin B on PC foil after scratch tests.

Scheme	Water Droplet		Ethylene Glycol Droplet	
	WCA [°]	CAH [°]	EGCA [°]	CAH [°]
PIL-A	143.3 ± 2.0	2.2 ± 1.4	137.3 ± 4.0	1.3 ± 1.1
PIL-B	127.4 ± 9.3	10.7 ± 0.6	109.1 ± 12.1	16.3 ± 8.0
C-RESS-A	135.8 ± 5.6	7.0 ± 5.7	135.5 ± 8.2	6.2 ± 2.1
C-RESS-B	138.8 ± 5.0	10.5 ± 1.7	121.9 ± 9.4	12.3 ± 2.4

## Data Availability

All results were analyzed by two independent observers. Data were measured at least two different locations on the sample and presented as mean value ± standard deviation (SD). All data included in this study are available upon request by contact with the corresponding author.
